# Immunomodulatory Effects of* Lactobacillus plantarum* Lp62 on Intestinal Epithelial and Mononuclear Cells

**DOI:** 10.1155/2016/8404156

**Published:** 2016-06-30

**Authors:** Thalis Ferreira dos Santos, Tauá Alves Melo, Milena Evangelista Almeida, Rachel Passos Rezende, Carla Cristina Romano

**Affiliations:** ^1^State University of Feira de Santana, Transnordestina Avenue S/N, 44030-900 Feira de Santana, BA, Brazil; ^2^State University of Santa Cruz, Highway Ilhéus-Itabuna, km 16 S/N, 45662-900 Ilhéus, BA, Brazil

## Abstract

Probiotic lactic acid bacteria are known for their ability to modulate the immune system. They have been shown to inhibit inflammation in experiments with animal models, cell culture, and clinical trials. The objective of this study was to elucidate the anti-inflammatory potential of* Lactobacillus plantarum* Lp62, isolated from cocoa fermentation, in a cell culture model. Lp62 inhibited IL-8 production by* Salmonella* Typhi-stimulated HT-29 cells and prevented the adhesion of pathogens to these epithelial cells. The probiotic strain was able to modulate TNF-*α*, IL1-*β*, and IL-17 secretion by J774 macrophages. J774 activation was reduced by coincubation with Lp62. PBMC culture showed significantly higher levels of CD4^+^CD25^+^ T lymphocytes following treatment with Lp62. Probiotics also induced increased IL-10 secretion by mononuclear cells.* L. plantarum* Lp62 was able to inhibit inflammatory stimulation in epithelial cells and macrophages and activated a tolerogenic profile in mononuclear cells of healthy donors. These results indicate this strain for a possible application in the treatment or prevention of inflammatory diseases.

## 1. Background

Probiotics are defined as live microorganisms which, when administered in adequate amounts, promote beneficial effects on the host's health. Microbial genera commonly associated with probiotic effects usually have the ability to restore the balance of microbiota, regulate intestinal traffic, produce short-chain fatty acids, and compete with pathogens for adhesion sites. Other properties, such as immune modulation and production of specific bioactive substances, are restricted to some strains. Traditionally, probiotics are used to treat or prevent the imbalance of the intestinal microbiota caused by pathogens and/or resulting from antibiotic therapy. However, new approaches have demonstrated the potential of these microorganisms as adjuncts to the treatment or prevention of intestinal and extraintestinal chronic diseases [1–3].

Inflammatory bowel diseases (IBD) have increased especially in western countries. Despite being considered to be caused by multifactorial conditions, the gut microbial population plays a central role in the development of IBD in genetically susceptible individuals [4]; therefore, therapeutic approaches that modify the local microbiota are very attractive. In this context, probiotics can stimulate the immune system, resulting in modulation of inflammatory mediators that are responsible for the maintenance of the pathological process or directing the innate and adaptive responses in a regulatory sense [5].

*L. plantarum* is a Gram-positive rod-shaped bacterium found in a wide variety of niches such as vegetables, meat, fish, and the gastrointestinal tract. Due to its ubiquity and importance in various fermentation processes, it was the first species of the genus* Lactobacillus* to have its genome sequenced. Further sequencing revealed considerable genetic diversity among strains isolated from different environments, which explains the high adaptability of these lactic acid bacteria [6]. A number of studies prove the applicability of various strains of* L. plantarum* as probiotic. The 299v strain, used in an already marketed probiotic, reduced* in vitro* expression of proinflammatory genes in a culture model of colonic mucosa [7]. In addition to anti-*Helicobacter pylori* activity [8], it was also able to improve the symptoms of irritable bowel syndrome in a clinical study using 200 patients [9].* L. plantarum* Lp91 showed strong immunoregulatory capacity in a murine colitis model induced by TNBS [10], and the WCFS1 strain was effective in generating regulatory T cells in healthy individuals [11].

The probiotic characteristics of each isolated strain are specific. Different species or variants within the same species can interact with the local microbiota and the host immune system in particular ways. Consequently, the use of* Lactobacillus* species as a probiotic needs careful selection to clarify their potential, mechanisms, and technological properties.* L. plantarum* Lp62 was isolated from a batch of fermenting cocoa beans and identified by 16S rDNA gene sequencing (GenBank access number KU291427). Its probiotic potential was attested previously in a study that evaluated its anti-inflammatory capacity in a colitis model induced by acetic acid in mice [12]. However, strain Lp62 was administered in a pool of other strains, making it difficult to establish the role of each microorganism in the observed effect. In this study, we sought to refine this research, by endeavoring to propose a possible* in vitro* anti-inflammatory mechanism. Strain Lp62 modulated the inflammatory response in epithelial cells by preventing* S.* Typhi adhesion, inhibited macrophage activation and thereby decreased the levels of cytokines involved in IBD pathogenesis, and, finally, increased IL-10 levels in mononuclear cells of healthy donors.

## 2. Materials and Methods

### 2.1. Cell Strains

HT-29 cells, a cell line derived from human colon adenocarcinoma, were cultured in 24-well plates, in DMEM (Gibco®) supplemented with 10% fetal bovine serum (Gibco) and 100 U·mL^−1^ penicillin and streptomycin, at an initial concentration of 10^6^ cells·mL^−1^, at 37°C and 5% CO_2_. The cultures were maintained for 15 d until the experiment day, and, during that period, the medium was replaced every two days.

The macrophage cell line J774A.1 (ATCC® TIB-67) was cultured at a concentration of 5 × 10^5^ cells·mL^−1^ in RPMI (Gibco) medium supplemented with 10% fetal bovine serum and 100 U·mL^−1^ streptomycin and penicillin, for 7 d in 5% CO_2_ and 37°C atmosphere, and the medium was replaced every two days until the experiment day.

Before inoculating microorganisms in the cell cultures, the medium was replaced with no added antibiotic.

### 2.2. Microorganisms

*L. plantarum* Lp62 was cultured in MRS medium (HiMedia) for 18 h at 37°C. The culture was then washed twice in 0.9% NaCl solution and used at a titer of 1 × 10^9^ CFU·mL^−1^.

*Salmonella enterica* serovar Typhi ATCC 6539 was cultured in Tryptic Soy Broth medium (HiMedia) for 18 h at 37°C, while stirring at 180 rpm. The culture was washed with 0.9% NaCl solution and diluted to reach *A*_600_ = 0.1, which corresponds to 10^8^ CFU·mL^−1^.

### 2.3. Separation of Peripheral Blood Mononuclear Cells (PBMCs)

Ten healthy donors were selected for blood collection. The group was composed of six men and four women, average age 26 years. Each individual took part in the study by signing the free informed consent term. The collection of blood from healthy donors was approved by the local ethics committee on human research (access number 106909), in accordance with guidelines established by the National Health Council. Blood was collected from donors in heparinized tubes and peripheral blood mononuclear cells were separated using Hystopaque® Sigma. 5 mL Hystopaque and 5 mL blood were added to a conical tube. After centrifuging at 400 ×g for 30 min, the mononuclear cells were collected and washed with RPMI. The concentration was adjusted to 5 × 10^5^ cells·mL^−1^ and the cells were then grown in RPMI supplemented with 10% fetal bovine serum at 37°C and 5% CO_2_.

### 2.4. Cytometry

For cytometric analysis, the cells were washed with PBS (2000 rpm, 10 min). To detect internal antigens, the cells were permeabilized using formaldehyde/saponin-based permeabilization IntraPrep*™* Kit (Beckman-Coulter). The macrophage lineage J774A.1 was externally labeled with anti-CD86-APC and anti-CD14-FITC. The HT-29 line was externally and internally labeled with anti-TLR-4-PE and anti-TLR2-FITC. Mouse IgG conjugated to FITC, PE, or APC was used as isotype control. PBMCs were externally labeled with anti-CD4-FITC, anti-CD25-PE, and intracellular anti-Foxp3-PE staining. Analyses were made in FC500 Beckman-Coulter cytometer. Data were processed using the Kaluza® flow analysis software.

### 2.5. ELISA

After bacterial cell coculture assays, the supernatants were collected for cytokine quantification by ELISA. The sandwich-ELISA procedures were performed according to the manufacturer's instructions. Kits for measurement of IL-8, IL-10, IL-1*β*, IL-12, IFN-*γ*, TNF-*α*, and IL-17 were obtained from PeproTech, Brazil.

### 2.6. Coculture Assays

An HT-29 cell culture was inoculated with* L. plantarum* Lp62 (10^9^ CFU·mL^−1^) and incubated for 2 h at 37°C and 5% CO_2_. Then, the wells were washed with PBS, inoculated with* S*. Typhi 6539 at a concentration of 10^8^ CFU·mL^−1^, and incubated for 2 h. In parallel,* S.* Typhi 6539 and Lp62 were added to HT-29 culture for 2 h, simultaneously. After cell-bacteria interaction, the supernatants were collected for cytokine assay. HT-29 cells were treated with trypsin-EDTA solution 0.25%, for cell detachment. The plates were incubated for 10 minutes at 37°C and then the trypsin was inactivated with fetal bovine serum. The cells were washed with RPMI and sent to flow cytometry. The effect of nonviable* Lactobacillus plantarum* Lp62 cells was also tested. Accordingly, a bacterial cell suspension was inactivated by heating at 80°C for 10 minutes. Cell viability was tested by plating on MRS medium. In addition, the proportion of adhering* S*. Typhi related to the initial inoculum was assessed by serial dilution and plating on MacConkey agar and the adherence percentage was calculated by the formula % adherence = CFU_final_/CFU_initial_ *∗*100. J774 cells were stimulated with 50 *µ*L* L. plantarum* Lp62 (0^9^ CFU·mL^−1^) and LPS (200 ng·mL^−1^) and incubated for 2 h at 5% CO_2_ and 37°C. PBMC cultures were similarly challenged, but the samples were incubated for 24 h. Supernatants were collected and J774 cells were detached by using cold RPMI. The cells were processed and analyzed by flow cytometry.

### 2.7. Data Analysis

The data shown represent the mean ± SD of the triplicate from three independent experiments. The statistical difference between the media (ANOVA) was assessed using GraphPad Prism 5.0 software.

## 3. Results and Discussion

The gastrointestinal tract mucosa is home to a diverse and large population of microorganisms. The epithelial layer and mucosa-associated immune system should be regulated in order to tolerate the resident microbiota and food antigens and simultaneously remain ready to respond to invasion of enteric pathogens. Accordingly, imbalance in the axis tolerance versus response leads to the development of a state of chronic intestinal inflammation, including ulcerative colitis (UC) and Crohn's disease (CD). Despite their peculiarities, inflammatory bowel diseases (IBD) are characterized by loss of epithelial barrier integrity, changes in expression level and spatial location of innate receptors, and increased production of proinflammatory cytokines [13]. In view of their effects on the immune response, probiotics have been used effectively in the treatment of gastrointestinal tract disorders. As the immune system is complex and compartmentalized, each probiotic strain interacts in a particular way, resulting in a specific response. In this study, we aimed at determining the anti-inflammatory effect of the* L. plantarum* Lp62 strain, testing its activity* in vitro* in a cell culture model.

Lp62 was isolated from cocoa pulp during seed fermentation. This strain was originally tested in a fermented milk drink containing other isolates from the same environment and was able to reverse chemically induced colitis in a nonisogenic animal model. However, the process of standardization, quality control, and industrial-scale production of multistrain probiotic formulations is quite laborious, so we prefer to focus studies on the strain with the most promising results. Initially, the Lp62 anti-inflammatory effect was tested on the HT-29 intestinal epithelial cell line and the pathogenic bacterium* S*. Typhi 6539 was used as an inflammatory stimulus. For this approach, the probiotic bacteria were added before adding the pathogen, or both were added simultaneously to the cell culture. After incubation, IL-8 production and the expression of Toll-like receptors 2 and 4 were evaluated. We also quantified pathogen adhesion to the epithelial cell in all treatments.

**Figure 1:**
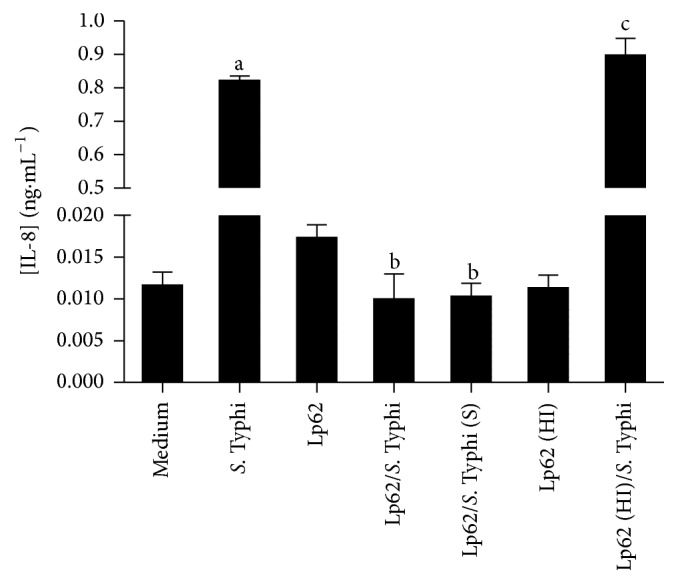
Quantification of IL-8 secreted by HT-29 in culture supernatant. HT-29 cells were treated with Lp62 and* S.* Typhi. Levels of IL-8 secreted into the culture medium were measured. Unstimulated cultures or cultures stimulated only with Lp62 or* S.* Typhi *i* were used as controls. S: inoculated simultaneously; HI: heat-inactivated.  ^a^Significant difference from the medium (without any stimulation).  ^b^Significant difference from* S*. Typhi-stimulated group.  ^c^Significant difference from Lp62/*S*. Typhi; *P* < 0.05.

**Figure 2:**
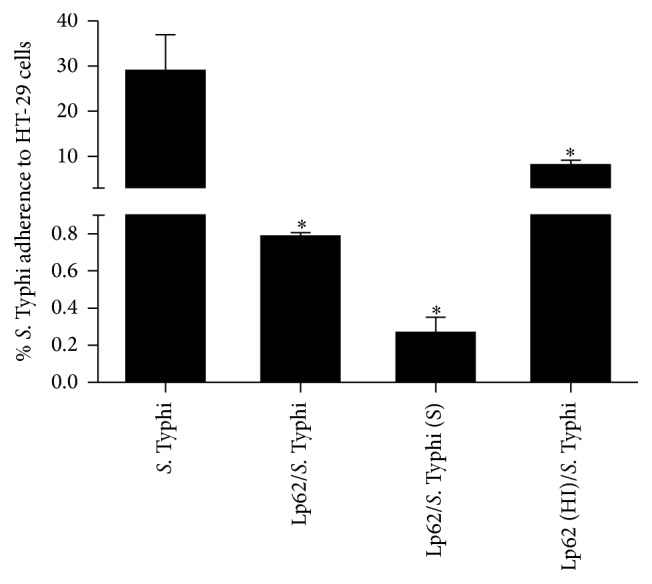
Percentage of* S*. Typhi adherence to HT-29 cells. HT-29 cells were treated with Lp62, and then* S*. Typhi was added to the culture. After incubation, the probiotic ability to inhibit pathogen binding to the epithelial cell was measured. The percentage of* S.* Typhi adherence was calculated in relation to the initial inoculum 1 × 10^8^. S: simultaneously inoculated; HI: heat-inactivated.  ^*∗*^Significant difference in relation to* S*. Typhi-stimulated group (*P* < 0.05).

*L. plantarum* Lp62 significantly reduced IL-8 production by HT-29 cells. In comparison with the control (0.8 ng·mL^−1^), which was only* S.* Typhi-stimulated, there was an approximately 80-fold reduction in both groups, treated with the probiotic prior to addition or simultaneously to the pathogen challenge (0.01 ng·mL^−1^). When the epithelial cell culture was stimulated with the probiotic alone, there was no significant cytokine production. Additionally, heat-inactivated Lp62 anti-inflammatory activity was investigated and it was observed that this group showed no decrease in IL-8 (±0.87 ng·mL^−1^), detected by ELISA (Figure 1). In accordance with these data, adherence of* S*. Typhi 6539 to HT-29 cells was statistically reduced in the groups treated with the probiotic Lp62 (Figure 2), showing that its anti-inflammatory action, in this model, may be related to the probiotic ability to prevent contact of the epithelial cell with the pathogen or competition for adhesion sites. Interestingly, the group treated with heat-inactivated probiotics had a higher percentage of pathogens attached to epithelial cells compared to other groups treated with probiotics, although it was significantly lower when compared to the control treated only with* S*. Typhi. This is probably the reason why this treatment has been unable to reduce IL-8 levels.

IL-8 is a chemokine that has chemoattractant activity, leading neutrophils to the site of the inflammatory stimulus. Like TNF-*α* and IL-1, it is expressed at high levels in the colonic mucosa of IBD patients [14]. The ability of probiotics to reduce* in vitro* IL-8 levels is well documented and serves as one of the basic parameters in the selection of probiotic bacteria with this potential. Ren et al. [15] observed a decrease in IL-8 produced by Caco-2 cells prestimulated by* L. plantarum* and challenged with* Salmonella *Typhimurium. In line with our findings, the probiotic caused strong inhibition of pathogen adhesion. The heat inactivation also led to loss of the anti-inflammatory effect. Carey and Kostrzynska [16] reported that preincubation with* Lactobacillus* and* Bifidobacterium* supernatant was able to inhibit IL-8 secretion by epithelial cells challenged with* S*. Typhimurium. The effect was lost when probiotics were inactivated by heat. As in the present study, these observations suggest that some factor released by metabolically active probiotic bacteria is responsible for the observed effect. We may thus emphasize that the inhibition of pathogen adhesion can contribute to the anti-inflammatory action.

**Figure 3:**
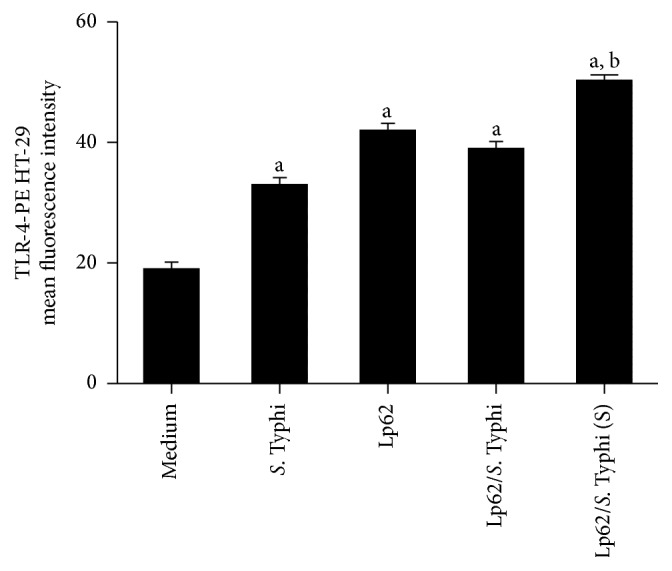
TLR-4 expression in HT-29 cells. HT-29 cells were stimulated with Lp62 and then challenged with* S*. Typhi. In parallel, the effect of simultaneous (S) addition of the two microorganisms was tested. HT-29 cells were labeled internally with anti-TLR-4 and analyzed by flow cytometry.  ^a^Statistically different from the medium (unstimulated cell).  ^b^Statistically different from the* S*. Typhi-stimulated group; *P* < 0.05.

No changes were detected in TLR2 expression in any experimental group. Interestingly, TLR-4 intracellular expression was found to be increased in Lp62-treated groups but did not differ significantly from the* S.* Typhi-stimulated control. When probiotic and pathogen were given simultaneously, there was a significant increase in receptor expression (Figure 3). LPS is a TLR-4 agonist. Under stimulation, the receptor triggers transcription of proinflammatory genes. In the intestinal mucosa, the receptors that recognize microbe-associated molecular patterns are expressed at low levels to avoid overstimulation and thus chronic inflammation. Alternatively, these receptors are expressed in a compartmentalized way, like TLR-5, which recognizes flagellin and is expressed basolaterally and is activated only if the colonic mucosa is invaded [17]. Despite its anti-inflammatory profile, Lp62 was able to raise TLR-4 expression; however, it was detectable only internally. According to Karlsson et al. [18],* L. plantarum* can be recognized by TLR-4, but, in our experiments, we believe that it was not able to activate the downstream route that leads to the production of proinflammatory cytokines such as IL-8. However, we did not investigate other products of TLR-4 activation in this cell model.

**Figure 4:**
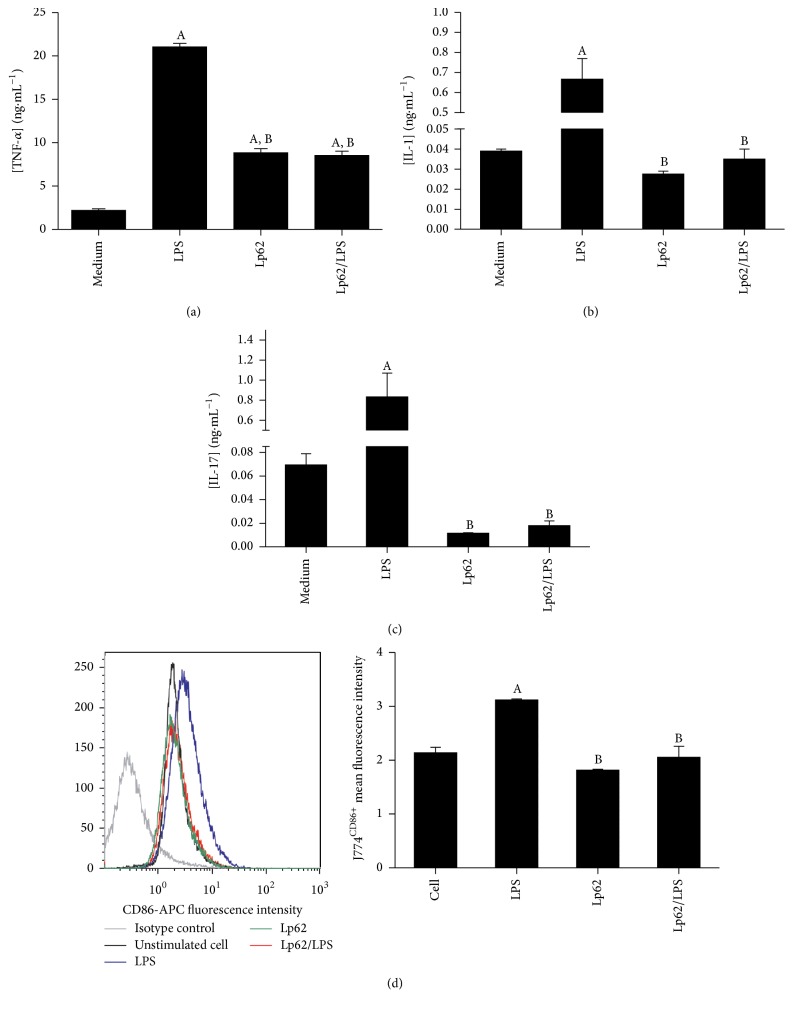
J774 macrophages stimulated with Lp62 and/or LPS: J774 macrophages were stimulated with LPS and Lp62 for 2 hours. The levels of IL-1, TNF-*α*, and IL-17 were measured in culture supernatant by ELISA ((a), (b), and (c), resp.). The CD86 expression was analyzed by flow cytometry (d).  ^A^Significant difference compared to unstimulated cells (culture medium).  ^B^Significant difference from the control stimulated with LPS only; *P* < 0.05.

Macrophages located in the intestinal lamina itself represent the major reservoir of these cells in the human body. They are adapted to efficiently remove any pathogen that tries to cross the mucosa, while maintaining homeostasis of the intestinal environment [19]. Considering that changes in the phenotypic and functional profile of these cells have implications in IBD pathogenesis, we decided to evaluate the Lp62 strain's capacity to inhibit the inflammatory stimulus in a J774 macrophage cell line. Secretion of TNF-*α*, IL-1*β*, IL-12, and IL-10 was measured in the cell culture supernatant and surface CD86 expression was evaluated by flow cytometry. J774 cell stimulation with LPS increased TNF-*α* IL-1 secretion 10 and 15 times, respectively (Figures 4(a) and 4(b)). Simultaneous cell challenge with Lp62 and LPS significantly decreased the secretion of these cytokines relative to the LPS control. Lp62 was also able to stimulate TNF-*α* release, but 2.5 times less than LPS-stimulated cell. Despite showing similar performance, with decreased secretion compared to treatment with Lp62, IL-12 showed no statistical difference between the groups. Likewise, the different treatments did not alter IL-10 levels secreted by J774 cells. Interestingly, LPS stimulated the release of high levels of IL-17 (±800 pg·mL^−1^), which was reversed by treatment with strain Lp62 (±10 pg·mL^−1^) (Figure 4(c)). A small but significant difference was detected in the costimulatory molecule CD86 expression on the J774 macrophage surface (Figure 4(d)). While incubation with LPS increased its expression, Lp62 or Lp62/LPS groups showed a reduction of activated macrophages.

Intestinal macrophages are adapted to maintain local homeostasis, even in a complex and potentially activating molecule-rich environment. However, in the inflamed mucosa, for example, in patients with CD and UC, macrophages exhibit an altered phenotype characterized by high expression of costimulatory molecules such as CD80 and CD86, as well as the innate receptors TLR-2 and TLR-4, specialized in detecting bacterial antigens [20, 21]. In this context, these cells become potent producers of proinflammatory cytokines such as IL-1*β*, TNF-*α*, IL-6, and MCP-1. Trials with murine and human cells have shown that probiotics can prevent or reverse the functional change of macrophages, characteristic of chronic inflammatory diseases. According to Pathmakanthan et al. [22],* L. plantarum* 299v reduced the secretion of TNF-*α* and IL-1*β* in mucosal mononuclear cells from IBD patients stimulated with* E. coli* or* Salmonella* Dublin and increased the IL-10 levels. TNF-*α* production is also affected by the LPS-stimulated macrophage RAW 2647 and treated with* Lactobacillus rhamnosus* GG [23]. Matsumoto and Benno [24] found that metabolites released in the stools of patients fed with yoghurt containing* Bifidobacterium animalis* LKM512 were able to reverse the inflammation caused by LPS in J774 cells. The effect of probiotic bacteria on antigens presenting cells such as macrophages and dendritic cells is strain-dependent, since they also may be able to upregulate the production of costimulatory molecules and proinflammatory cytokines [25]. IL17 induces neutrophil recruitment to the inflamed site and triggers the release of inflammatory cytokines in macrophages. However, its role in inducing colitis remains uncertain, as it even presents a protective activity in the gut, depending on the model studied. The main source of this cytokine is Th17 cells; however, the innate immunity cells, including macrophages, can produce it [26]. Here, we observe that Lp62 modulated IL-17 secretion in J774 macrophages. Further studies are needed to determine the impact of this probiotic on IL-17 production in the* in vivo* colitis model. In the present study, we speculate that* L. plantarum* Lp62 was capable of limiting J774 macrophage activation and consequently preventing proinflammatory cytokine secretion, contributing to the maintenance of local homeostasis.

**Figure 5:**
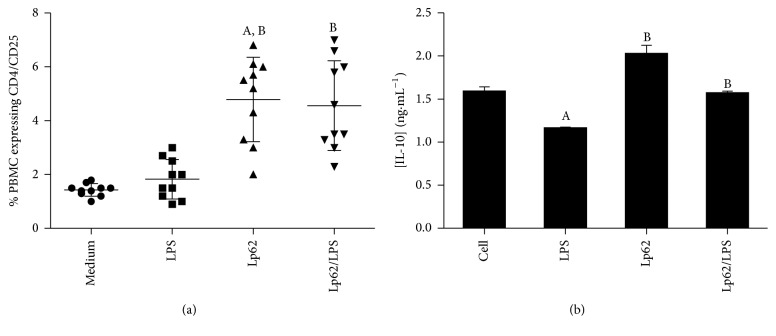
CD4^+^CD25^+^ T lymphocytes and IL-10 secretion in PBMC treated with Lp62. Cultures of peripheral blood mononuclear cells were challenged with LPS and Lp62. The proportion of CD4^+^CD25^+^ cells was determined by flow cytometry (a). IL-10 production was examined in the culture supernatant by ELISA (b).  ^A^Statistical difference compared to the control without stimulation.  ^B^Statistical difference from the control only stimulated with LPS; *P* < 0.05.

On the way to elucidate the Lp62 anti-inflammatory profile, its ability to induce a regulatory phenotype in systemic circulation lymphocytes was checked by analyzing the CD4^+^CD25^+^Foxp3^+^ population and the IL-10 secretion by peripheral blood mononuclear cells. Lp62 stimulated PBMC presented CD4^+^CD25^+^ population around 5%, significantly different from the unstimulated control and the control stimulated with LPS alone. Incubating cells with Lp62/LPS increased the percentage of this population significantly compared to the unstimulated control (Figure 5(a)). However, no differences were found in intracellular staining of Foxp3 between groups. Lp62 displayed the ability to increase IL-10 production in PBMCs. The IL-10 level from the Lp62/LPS treated group also was significantly increased as compared to the unstimulated control. PBMC challenged with LPS only showed lower IL-10 secretion compared to the other groups (Figure 5(b)).

IL-10 producing regulatory T cells can be found in the intestinal mucosa of healthy humans and mice. In studies involving the transfer of Treg cells, IL-10 produced by these cells were able to attenuate colitis [27]. The ability of some probiotic strains to activate a regulatory profile is well documented in clinical trials. According to Dong et al. [28], feeding with* L. casei* Shirota for 4 weeks increased the IL-10/IL-12 ratio in the plasma of healthy individuals and the expression of CD25 on T cells was significantly higher. IL-10 serum levels were higher after consumption of* Lactobacillus salivarius* CECT5713 [29]. Similar to our findings, the mixture of* L. plantarum* CECT 7315 and CECT 7316 was able to raise the percentage of T lymphocytes CD4^+^CD25^+^ and IL-10 mucosal levels [30]. Strain Lp62 increased the population of CD4^+^CD25^+^ lymphocytes in PBMC culture, but significant expression of Foxp3 was not detected. Treg cells are characterized by CD4^+^CD25^+^ expression on the surface but are dependent on the Foxp3 transcription factor to exercise their function on colonic* lamina propria*. Increased IL-10 levels after treatment with Lp62 point to a regulatory T cell profile, but cytokine production by other cells present in the culture should be considered.

In the intestinal environment, epithelial cells, microorganisms, and immune cell aggregates contribute to maintaining homeostasis. According to the widely accepted model, epithelial cells are responsible for releasing factors that will direct the antigen presenting cells to a nonresponsive profile or activating a regulatory response. The T cells generated in this environment would be responsible for maintaining homeostasis by releasing considerable amounts of IL-10 and TGF-*β*. Evidence suggests that the composition of the local flora is directly correlated to the balance between response and tolerance. In this sense, probiotics have been effective in restoring the tolerogenic profile of the intestinal mucosa, by modulating the activity of the cells that participate in this process [17, 19, 27]. In this paper, the marked anti-inflammatory effect related to the lactic acid bacteria* L. plantarum* Lp62 was observed on intestinal epithelial cells, macrophage, and lymphocyte. In a cell culture model, this strain was able to prevent* S*. Typhi adhesion to epithelial cells and hence inhibit IL-8 secretion. A slight decrease in macrophage activation was also observed which may have contributed to reducing proinflammatory cytokine production. Finally, the Lp62 strain was able to enhance IL-10 secretion and increase the CD4^+^CD25^+^ cell population. Since it showed immunomodulatory capacity on the main cells involved in the intestinal mucosal immunity, Lp62 is a strong candidate to assist in therapy for inflammatory diseases.

## 4. Conclusions

The results presented in this paper should serve as a basis for further studies that can investigate the pathways involved in the Lp62 anti-inflammatory effect. Equally important are approaches in search of safe use of all the newly discovered strains, mainly because probiotics are used in the context of a previously damaged mucosa. Furthermore,* in vivo* trials are essential in the study of probiotic action due to particularities and the high complexity of the intestinal environment.

## Supplementary Material

The additional material shows the detection of TLR-4 in HT-29 cells by flow cytometry. A representative histogram of each experimental group is shown. The values of the average fluorescence intensity are displayed as a bar graph in the article body (Figure 3).
