# Supplementary material for: Immunomodulatory Effects of Lactobacillus plantarum Lp62 on Intestinal Epithelial and Mononuclear Cells
Source: Biomed Res Int. 2016 Jun 30;2016:8404156. doi: 10.1155/2016/8404156 (PMC4944036; doi:10.1155/2016/8404156)
Supplement: Supplementary file 1 — The additional material shows the detection of TLR-4 in HT-29 cells by flow cytometry. A representative histogram of each experimental group is shown. The values of the average fluorescence intensity are displayed as a bar graph in the article body (Figure 3). [file 8404156.f1.pdf]

Fig 3 - supplementary figure

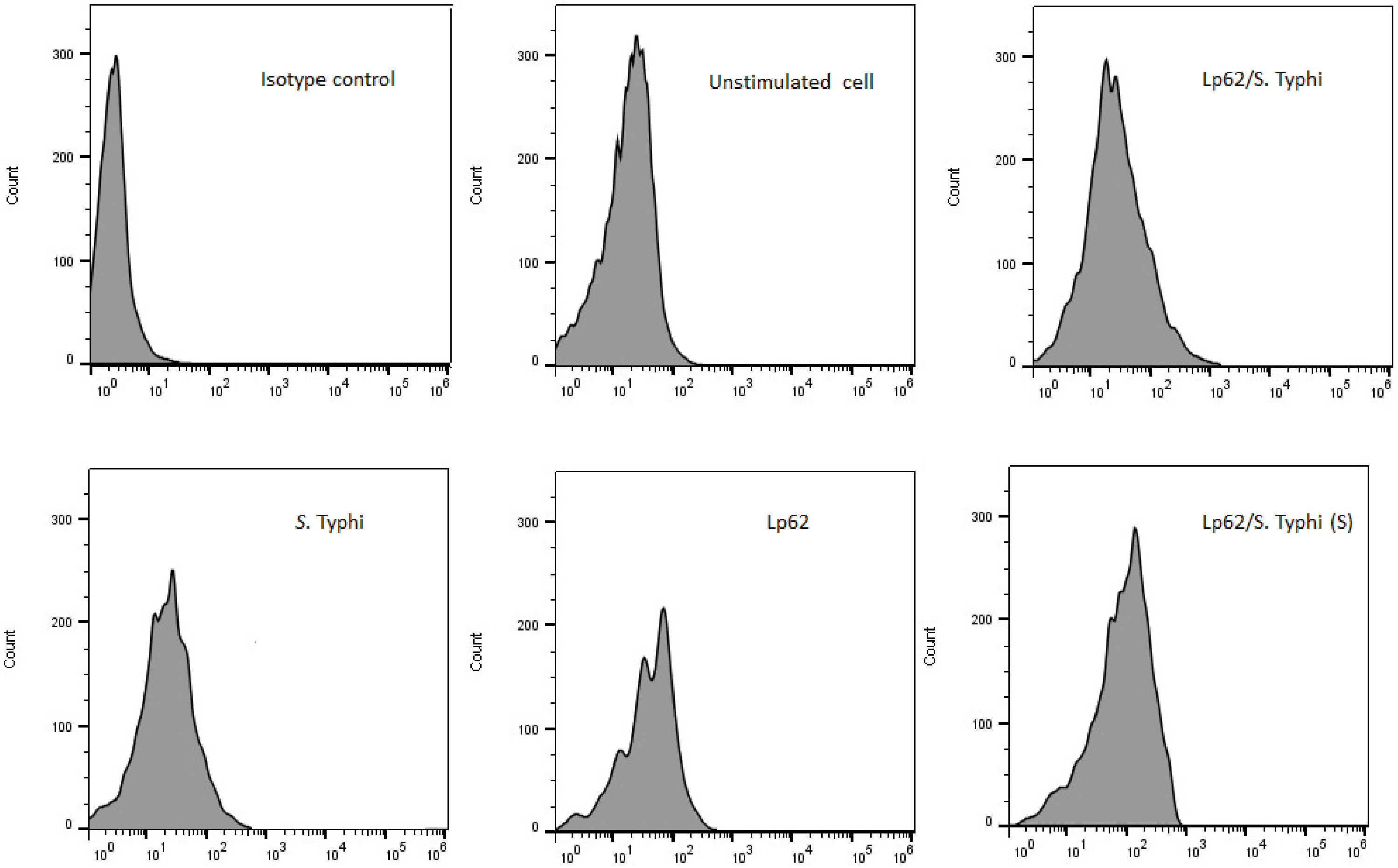

Fig 3. TLR-4 expression in HT-29 cells. HT-29 cells were stimulated with Lp62 and then challenged with *S*. Typhi. In parallel, the effect of simultaneous (S) addition of the two microorganisms was tested. HT-29 cells were labeled internally with anti-TLR4 and analyzed by flow cytometry.
